# Telehealth for Parkinson disease patients during the COVID-19 pandemic: the TeleParkinson study

**DOI:** 10.1055/s-0042-1758751

**Published:** 2022-12-19

**Authors:** Danielle Pessoa Lima, Vlademir Carneiro Gomes, Antonio Brazil Viana Júnior, Francisco Mateus Carvalho de Assis, Pedro Henrique Avelino Oliveira, Letícia Chaves Vieira Cunha, Isabelly Cavalcante Braga, Miriam Lindsay Silva Marques, Jézica de Sousa Assunção, Adeline Louise Lopes Damasceno, Ana Lara Guerra Barbosa, Arthur Holanda Moreira, Maria Eduarda Quidute Arrais Rocha, Maria Eduarda Mendes Pontes Porto, Érica Carneiro Barbosa Chaves, Liliane Maria de Oliveira, Jarbas de Sá Roriz Filho, Manoel Alves Sobreira Neto, Pedro Braga Neto

**Affiliations:** 1 Universidade Federal do Ceará, Hospital Universitário Walter Cantidio, Departamento de Clínica Médica, Divisão de Geriatria, Fortaleza CE, Brazil.; 2 Universidade de Fortaleza, Centro de Ciências da Saúde, Fortaleza CE, Brazil.; 3 Universidade Federal da Paraíba, Departamento de Educação Física, João Pessoa PB, Brazil.; 4 Universidade Federal do Ceará, Hospital Universitário Walter Cantidio, Centro de Pesquisa Clínica, Fortaleza CE, Brazil.; 5 Universidade Federal do Ceará, Instituto de Educação Física e Esportes, Fortaleza CE, Brazil.; 6 Universidade Federal do Ceará, Departamento de Fisioterapia, Fortaleza CE, Brazil.; 7 Universidade Federal do Ceará, Faculdade de Medicina, Fortaleza CE, Brazil.; 8 Universidade Federal do Ceará, Departamento de Biologia, Fortaleza CE, Brazil.; 9 Centro Universitário Estácio do Ceará, Departamento de Educação Física, Fortaleza CE, Brazil.; 10 Universidade Unichristus, Faculdade de Medicina, Fortaleza CE, Brazil.; 11 Universidade Federal do Ceará, Hospital Universitário Walter Cantidio, Departamento de Clínica Médica, Divisão de Neurologia, Fortaleza CE, Brazil.

**Keywords:** Parkinson Disease, Feasibility Studies, Telemedicine, Sleep, Doença de Parkinson, Estudos de Viabilidade, Telemedicina, Sono

## Abstract

**Background** Telemedicine allows Parkinson disease (PD) patients to overcome physical barriers to access health care services and increases accessibility for people with mobility impairments.

**Objective** To investigate the feasibility indicators of a telehealth intervention for PD patients, including patient recruitment, attendance, technical issues, satisfaction, and benefits on levels of physical activity and sleep.

**Methods** We conducted a single-center, single-arm study of telehealth video consultations using WhatsApp (Meta Platforms, Inc., Menlo Park, CA, USA). Also, we collected the feasibility indicators as the primary endpoints. All the patients in the study were previously evaluated in person by the same team.

**Results** Patient recruitment, attendance, and technical issues rates were 61.3%, 90.5%, and 13.3%, respectively, with good scores of patient acceptance and satisfaction with the study intervention. The telehealth intervention improved physical activity, including the number of walks for at least 10 continuous minutes ( *p* = 0.009) and the number of moderate-intensity activities lasting at least 10 continuous minutes ( *p* = 0.001). The Pittsburgh sleep quality index (PSQI) scores also improved for one of its components: perceived sleep duration ( *p* < 0.001) and for total Pittsburgh score ( *p* < 0,001). The average travel time saving was 289.6 minutes, and money-saving was R$106.67 (around USD 18; almost 10% of the current minimum wage in Brazil).

**Conclusions** Direct-to-patient telehealth video consultations proved to be feasible and effective and had a positive impact on physical activity levels and sleep in PD patients.

## Resumo

**Antecedentes** A telemedicina permite que pacientes com doença de Parkinson (DP) superem barreiras físicas para acessar serviços de saúde e aumenta a acessibilidade para pessoas com mobilidade reduzida.

**Objetivo** Investigar indicadores de viabilidade de uma intervenção em telessaúde para pacientes com DP, incluindo recrutamento, atendimento, aderência, problemas técnicos, satisfação e benefícios nos níveis de atividade física e sono.

**Métodos** Foi conduzido um estudo de centro e braço únicos baseado em consultas por telessaúde com utilização do WhatsApp (Meta Platforms, Inc., Menlo Park, CA, EUA). Foram calculados indicadores de viabilidade como desfechos primários.

**Resultados** As taxas de recrutamento, atendimento e problemas técnicos foram 61,3%, 90,5% e 13,3%, respectivamente, com bons escores de aceitação e satisfação com a intervenção. A intervenção melhorou os níveis de atividade física, incluindo o número de passos por pelo menos 10 minutos contínuos ( *p* = 0,009) e o número de atividades intensas e moderadas com duração de pelo menos 10 minutos contínuos ( *p* = 0,001). O Índice de Qualidade do Sono de Pittsburgh melhorou nos seguintes componentes: duração percebida do sono ( *p* < 0,001) e escore total ( *p* < 0,001). A média do tempo de viagem médio poupado foi de 289,6 minutos, e a economia financeira foi de R$ 106,67 reais (por volta de USD 18; quase 10% do salário mínimo atual do Brasil).

**Conclusões** As consultas por vídeo provaram ser viáveis e efetivas, com impacto positivo nos níveis de atividade física e sono de pacientes com DP.

## INTRODUCTION

The coronavirus disease 2019 (COVID-19) pandemic imposed several restrictions measures to reduce severe acute respiratory syndrome coronavirus 2 (SARS-CoV-2) infection, including the postponement of elective medical procedures and outpatient consultations. 1 Then, Brazil's government exceptionally issued a new decree authorizing the practice of telemedicine during the pandemic. 2

Telemedicine allows patients to overcome physical barriers to access health care services and increases accessibility for people with mobility impairments, such as Parkinson disease (PD) patients, and those living in areas with minor access to health services. 3 Studies have shown similar effectiveness of virtual consultations versus face-to-face appointments to address symptoms with higher satisfaction rates. 3 4 5

Brazil's public health system faced enormous challenges during the COVID-19 pandemic. Thus, this study aimed to assess feasibility indicators of a telehealth intervention for PD patients. We hypothesized that the virtual visits would be beneficial and viable due to saving money and time, especially during the context of the COVID-19 pandemic due to limited access to medical care during the period of isolation.

## METHODS

### Investigar

We conducted a single-center, single-arm study to assess feasibility indicators as primary endpoints (patient recruitment, attendance, technical issues rates, satisfaction, travel time and money savings, and benefits) of virtual medical consultations.

The study was conducted at the Department of Neurology of Hospital Universitário Walter Cantídio (HUWC) in Fortaleza, Brazil, from May 1 ^st^ to December 31 ^st^ , 2020. The study was approved by the HUWC ethics committee (registration number 31232720.2.0000.5045) and the Brazilian Trial Registry (REBEC) RBR-6pq44p. Also, all patients signed a written informed consent form.

### Study participants

We have consecutively drawn the patients from the outpatient clinic's medical appointment scheduling list. The patients were invited in a phone call to participate. We screened those who agreed to participate for eligibility. If eligible, we requested them to attend a remote medical consultation using WhatsApp (Meta Platforms, Inc., Menlo Park, CA, USA) video calls. We chose this tool because our patient population is familiar with this technology in their daily lives.

The eligibility criteria included the diagnosis of PD according to the United Kingdom Parkinson's Disease Society Brain Bank 6 and receiving care at our outpatient clinic in the preceding 12 months. We excluded those who were uncomfortable with virtual medical consultation, those who did not have access to communication technology, and patients with dementia whose caregivers were not available to give us the necessary information about their clinical complaints.

### Study intervention

All teleconsultations were held once a week during afternoon hours following an interview procedure, like face-to-face consultations. The study intervention consisted of teleconsultation followed by health education. The health education material and oral recommendations are not part of the regular in-person visit routine. Several steps were involved in the intervention: taking a list of patients scheduled for recruitment weekly, collecting outcomes data of pre- and post-teleconsultation sleep and physical activity questionnaires, interviewing about the type of transportation, time and money spent on face-to-face consultations, dialogue, and demonstration of health education in a Whatsapp video call, sending educational material, calling 15 days after the teleconsultation to reinforce recommendations, and organizing prescriptions and additional requests to send to patients.

A geriatrician with movement disorders training conducted the consultations. A multidisciplinary team from the Living with Parkinson's Disease Research and Extension Project at the Universidade Federal do Ceará performed the health educational approach. This team was composed of health professionals and students from the following areas: biological sciences, physical education, physiotherapy, and medicine. We used visual impressions and tests to conduct the physical examination, which included assessing tremor, bradykinesia, gait, speaking function, and dyskinesia. As the appointment was online, we did not evaluate the stiffness, cardiopulmonary auscultation, and blood pressure.

In the final part of the teleconsultation, our team made some health-related recommendations. We constructed the health education material, and the team explained the content, demonstrating the exercises via WhatsApp video. Also, we sent the material in portable document format (PDF) by WhatsApp text message. The patients with mild-to-moderate disease received advice on healthy eating, sleep hygiene, physical activity, fall prevention, and non-pharmacological management approaches for urinary incontinence. The participants were encouraged to engage in regular physical activities for at least 30 minutes 3 times a week. We emphasized the need to reduce sedentary time by incorporating multiple short breaks at least every 60 minutes during continuous bouts of sedentary behavior.

The patients were taught to follow instructions about a healthy sleep routine and train their brains and bodies to receive the full amount of sleep they required. Techniques to prioritize sleep and maintain consistency in their routines were discussed, such as having a fixed get-up time, whether it is on weekdays or weekend, not napping too much during the day, avoiding substances that may cause sleep interruptions (like caffeine, nicotine and alcohol late in the afternoon as well as heavy meals), and unplug electronic devices as cell phones, tablets, and computers (as they may reduce the melatonin synthesis). They were also instructed to be physically active, to obtain daylight exposure, since sunlight is a crucial promoter of circadian rhythms, and trying relaxation methods that might help prepare them to sleep, including meditation, mindfulness, and timed breathing.

Those with severe PD and limited mobility received advice on healthy nutrition, sleep hygiene education (SHE), approaches to reduce the risk of aspiration pneumonia and to prevent skin lesions, contractures, and pain. We sent health education materials and medication schedules to improve adherence ( Supplementary Tables 1 and 2 ).

**Table 1:** Baseline demographic and clinical characteristics of the study participants

Variable		Values
Sex	Male	63 (58.9%)
	Female	44 (41.1%)
Age		65.7 ± 17.1
Education	Illiterate	10 (9.3%)
	Incomplete primary education (1–8 years)	48 (44.9%)
	Complete primary education (9 years)	7 (6.5%)
	Incomplete secondary education (10–11 years)	3 (2.8%)
	Complete secondary education (12 years)	20 (18.7%)
	Incomplete tertiary education	7 (6.5%)
	Complete tertiary education	7 (6.5%)
	Graduate education	5 (4.7%)
Disease duration		10 ± 6.7
Antiparkinsonian drugs	Levodopa	101 (94.4%)
	Pramipexole	51 (47.7%)
	Amantadine	27 (25.2%)
	Entacapone	25 (23.4%)
	Rasagiline	15 (14%)
	Extended-release levodopa	32 (29.9%)
	Levodopa equivalent dose	1060.5 ± 880.4
Medications used	Antihypertensive drugs	45 (42.1%)
	Antidepressants	44 (41.1%)
	Benzodiazepine	22 (20.6%)
	Statins	23 (21.5%)
	Vitamin D	23 (21.5%)
	Calcium	19 (17.8%)
	Oral antidiabetics	22 (20.6%)
	Atypical antipsychotics	14 (13.1%)
	Bisphosphonate	12 (11.2%)
Number of medications used		5.9 ± 3
Visual hallucination		34 (32.7%)
Motor fluctuation		65 (63.1%)
Urge incontinence		44.7%
Constipation		37 (35.2%)
Impulse-control disorders		22 (21.2%)
SE ADL		70.3 ± 25.8
Dyskinesia		51 (49%)

Abbreviation: SE ADL, Schwab & England activities of daily living.

Note: Data expressed in frequency (percentage) or mean ± standard deviation (median).

**Table 2:** Medical interventions adopted in the TeleParkinson study

Variables	Values
Antiparkinsonian drug adjustments	63 (58.9%)
Request of laboratory tests	65 (60.7%)
Vitamin D supplementation	38 (35.5%)
Physical rehabilitation care	31 (29.0%)
Laxative medications	31 (29.0%)
Antidepressant medications	23 (21.5%)
Pharmacological treatment of insomnia	20 (18.7%)
Request of bone densitometry	34 (31.8%)
Calcium supplementation	22 (20.6%)
Request of brain imaging	17 (15.9%)
Pharmacological treatment of urge incontinence	14 (13.1%)
Average duration of teleconsultation	64.33 ± 23.97

Note: Data expressed in mean ± standard deviation (median) or frequency (percentage).

Remote reassessment was determined by the attending geriatrician or at the patient's/caregiver's request. We virtually reassessed patients who needed earlier reassessment due to prescription changes or clinical complications. The decision of whether the reassessment should be remote or in person depended on the patient complaint and its gravity. We used the Research Electronic Data Capture (REDCap) software (Vanderbilt University, Nathville, TN, USA) for data collection and management.

### Sample size

The study sample was drawn consecutively from a population of 350 PD patients attending our outpatient clinic who meet the eligibility criteria, 4 patients per week. We established the recruitment period based on logistics and financial considerations.

### Characterization measures

We collected the data through a chart review and patient questionnaires. We defined the levodopa equivalent dose (LED) of an antiparkinsonian drug as the dose that produces the same level of symptomatic control as 100 mg of immediate-release L-dopa. 7 We used the Schwab and England Activities of Daily Living (SE ADL) scale. We questioned the participants about functional decline due to social restriction measures imposed during the pandemic.

We administered three questionnaires before and between 30 and 45 days after the telehealth intervention to assess its benefits, and the following questions to evaluate patient adherence to treatment (i.e., to what extent did the patient adhere to medical recommendations such as medications and new management approaches?) and health recommendations (i.e., has the patient been engaging in physical activity at least 30 minutes 3 times a week?).

The three questionnaires were validated for the Brazilian population and were as follows: the Pittsburgh Sleep Quality Index – PSQI 8 ; the Epworth Sleepiness Scale – ESS 9 ; and the International Physical Activity Questionnaire – Short Form - IPAQ-SF. 10 The IPAQ-SF is an instrument recommended by the United States Center for Disease Control and Prevention to assess self-reported physical activity. We classified the respondents as sedentary, insufficiently active A, insufficiently active B, active, and very active. 10

### Feasibility indicators

We calculated the recruitment rate by the number of patients who agreed to participate in the study divided by the number of patients contacted. We obtained the attendance rate by the proportion of virtual consultations completed as scheduled. We considered the intervention feasible if at least 80% of the consultations were entirely done based on previous studies of telemedicine. In 2017, a national randomized controlled study of virtual house calls was performed in 5 states in the United States to determine whether this model of specialty care delivery is feasible. 4 Virtual house calls were considered feasible if 80% of participants in the intervention arm completed at least one visit and if at least 80% of virtual visits were completed as scheduled. Also, the *Virtual visits for Parkinson disease* study evaluated participants from a multicenter cohort in the United States and defined its feasibility by the proportion of visits completed as scheduled with at least 80% of completion. They considered that the visits were acceptable to PD patients if at least 80% were interested in future virtual visits for their illness. 11 Achey et al. 12 and Dosey et al. 13 also evaluated the feasibility of telemedicine for patients with PD as primary outcome, and prespecified a threshold of 80% of the completed visits.

We provided some instructions to optimize the remote consultation: choose an adequate room with adequate wi-fi signal, have a caregiver or family member present during the entire appointment for those with dementia, and have the most recent blood test results and the list of medications at hand during consultation.

Patients answered a self-administered questionnaire to evaluate whether the telehealth intervention was safe, feasible, satisfying, and well-accepted for future use. In cases in which the patients had dementia, the caregiver answered the questions.

**Figure 1.**
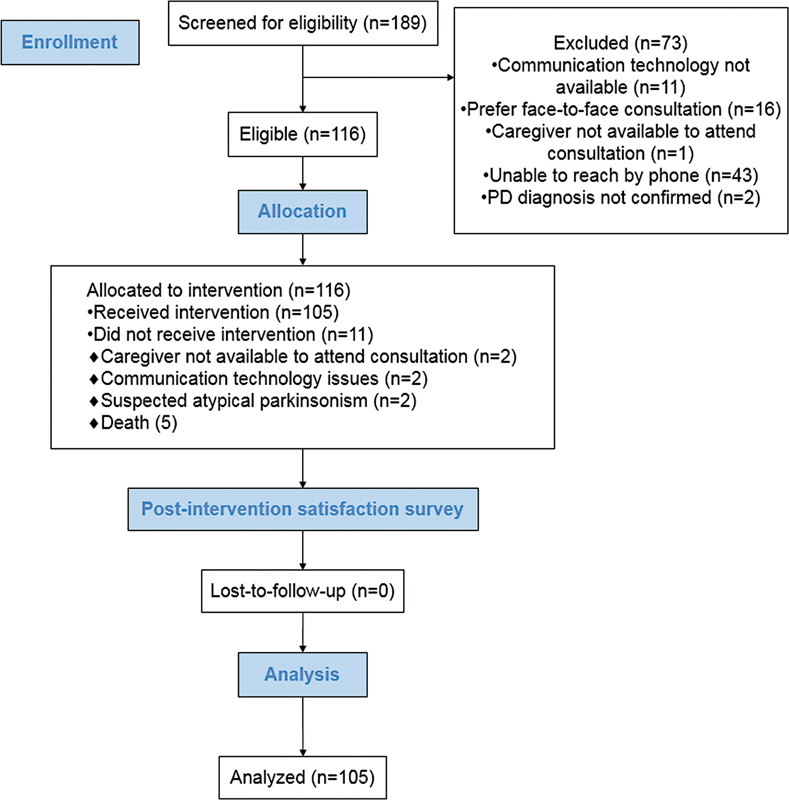
Box plot for satisfaction.

The questionnaire consisted of 10 questions with a visual analog scale (VAS) to evaluate satisfaction (worst on the left end and best on the right end). Each question uses a graphical scale with five satisfaction faces. We requested the participants to mark the point that was a representation of their level of satisfaction. They could only select one answer. A score of 75 indicated satisfaction (i.e., easy; they were satisfied and felt safe). A score of 100 indicated higher satisfaction (i.e., very easy; they were very satisfied and felt very safe) ( Figure 1 ).

The questionnaire has five domains: feasibility (items 1–2); a sense of safety (items 3–4); satisfaction (items 5–6); effectiveness (items 7–8); and a future use of the intervention (items 9–10). The VAS scale was sent on WhatsApp as a REDCap link.

We calculated the rate of technical issues during consultations by dividing the number of virtual visits with internet connection issues by the number of consultations. We asked patients about transportation, average travel time from the clinic to their home, and travel expenses associated with a face-to-face consultation.

### Statistical analysis

For numerical variables, we presented data as means, standard deviations, and medians. For categorical variables, we described the data as frequencies. To investigate the existence of an association between the variables, we used the Mann-Whitney and McNemar tests. We built box plots to demonstrate the distribution of satisfaction scores and adopted a significance level of 5%. All statistical analyses were performed using the Jamovi 1.8 software.

## RESULTS

**Figure 2.**
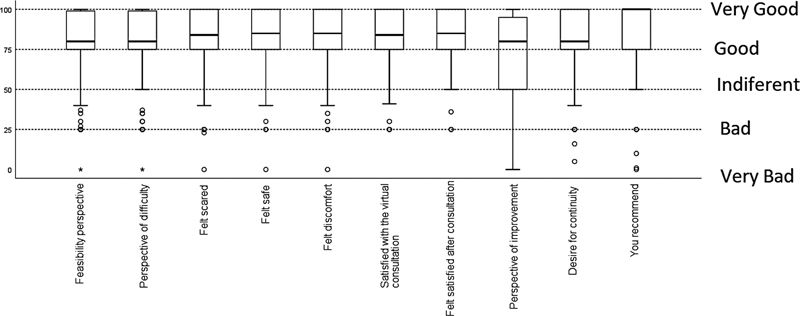
TeleParkinson study flowchart.

We contacted the first 191 patients from the clinic's medical appointment scheduling list, and 118 agreed to participate. The recruitment rate was 61.7%, and the attendance rate was 90.7%. Figure 2 shows the study flowchart.

We selected 118 patients for the intervention, but 11 of them did not receive it because of caregiver unavailability (n = 2), communication technology unavailability (n = 2), PD diagnosis not completely defined (n = 2), and death (n = 5). Seventeen (15.8%) patients requested remote reassessments. The reasons for reassessment included motor fluctuations (n = 14), pain control (n = 1), depression treatment optimization (n = 1), and sleep disturbances (n = 1). Eight (7.4%) patients were advised to come to the clinic for a face-to-face consultation because they required physical examination. Technical issues were evidenced during the teleconsultation for 12 (11.2%) patients due to internet connection problems.

Table 1 describes the demographic characteristics of our sample. Most participants were male, with a mean age of 65.7 ± 17.1 years old and mean disease duration of 10.0 ± 6.7 years. A small proportion (8.6%) was attending physical therapy sessions; 25.7% engaged in physical activity for at least 30 min, 3 times per week, and 36.5% needed walking aids. Thirty-five patients (32.7%) reported perceived functional decline and worsening of parkinsonian symptoms during the pandemic.

Table 2 describes the medical interventions adopted in the teleconsultation. Also, we requested laboratory tests, bone densitometry, physical rehabilitation care, and brain imaging. Table 3 shows the patient's adherence to non-pharmacological and pharmacological approaches.

**Table 3:** Patient adherence to TeleParkinson intervention

Variable	n (%)
Patient adherence to nonpharmacological health recommendations	
1	20 (20.4%)
0.75	33 (33.7%)
0.5	33 (33.7%)
0.25	9 (9.2%)
0	3 (3.1%)
Patient adherence to prescribed medications	
1	62 (63.3%)
0.75	24 (24.5%)
0.5	6 (6.1%)
0.25	2 (2%)
0	4 (4.1%)
Are patients exercising for at least 30 minutes, 3 times a week, post intervention?	
Yes	58 (59.2%)
No	40 (40.8%)

Note: Data expressed in mean ± standard deviation (median) or frequency (percentage).

The impact of the intervention on sleep and levels of physical activity are described in Tables 4 and 5 . There were improvements in PSQI total score and in one of its component : sleep duration (component 3). As for the level of physical activity, there was an increase in the number of days of walking for at least 10 continuous minutes and the number of days of moderate activities for at least 10 minutes continuously ( Table 5 ). Also, the satisfaction with remote consultations is shown in Figure 1 .

**Table 4:** Effect of TeleParkinson in sleep questionnaires

Variable	Baseline	Post virtual appointment	*p*	ES
PSQI total score	12.1 ± 3.6 / 12 (9–15)	10.7 ± 3.6 / 11 (8–13)	< 0.001a	d = 0.44
Component 1- Sleep quality	1.5 ± 0.8 / 1 (1–2)	1.4 ± 0.8 / 1 (1–2)	0.216b	r = 0.24
Component 2- Sleep onset latency	1.9 ± 1 / 2 (1–3)	1.6 ± 1.3 / 2 (0–3)	0.067b	r = 0.28
Component 3- Sleep duration	1.7 ± 1.3 / 2 (0–3)	1.1 ± 1.3 / 1 (0–3)	< 0.001b	r = 0.63
Component 4 - Sleep efficiency	2.9 ± 0.6 / 3 (3–3)	2.9 ± 0.4 / 3 (3–3)	0.299b	r = 0.43
Component 5 - Sleep disorders	2.3 ± 0.8 / 2 (2–3)	2.1 ± 0.7 / 2 (2–3)	0.066b	r = 0.31
Component 6 - Hypnotic drug use	1.5 ± 1.5 / 1 (0–3)	1.3 ± 1.4 / 0 (0–3)	0.607b	r = 0.12
Component 7- Daytime repercussion	1.7 ± 1 / 2 (1–3)	1.7 ± 1 / 1 (1–3)	0.532b	r = 0.10
ESS score	11.2 ± 5.9 / 11 (6–16)	10.1 ± 7.4 / 8 (3–17)	0.567b	d = 0.09

Abbreviation: PSQI, Pittsburgh Sleep Quality Index; Notes: Data expressed as mean +/− standard deviation, median (25th percentile − 75th percentile). a: Teste t de Student; b: Teste de Mann-Whitney; d: Cohen d; r: Rank Biserial Correlation.

**Table 5:** Effect of TeleParkinson in physical activity questionnaire-short version

PAQ- SV	Baseline	Post virtual appointment	*p*	ES
1a. How many days in the last week did you WALK for at least 10 continuous minutes at home or at work, as a form of transportation to get from one place to another, for leisure, for pleasure or as a form of exercise? (Say the number of days)	2.6 ± 2.8 / 2 (0–5)	4 ± 3.8 / 4 (0–7)	0.009	0.42
1b. On days when you walked for at least 10 continuous minutes, how much time in total did you spend walking each day? (In minutes)	18.5 ± 29.1 / 6 (0–30)	17.7 ± 19.4 / 15 (0–30)	0.888	0.02
2a. On how many days in the past week, have you performed MODERATE activities for at least 10 continuous minutes, such as cycling lightly on the bike, swimming, dancing, doing light aerobics, playing recreational volleyball, carrying light weights, doing housework at home, at yard or garden like sweeping, vacuuming, gardening, or any activity that moderately increased your breathing or heartbeat (PLEASE DO NOT INCLUDE WALK) (Say the number of days)	2.1 ± 2.7 / 0 (0–4)	3.5 ± 2.7 / 3 (0–7)	0.001	0.49
2b. On the days that you have done these moderate activities for at least 10 continuous minutes, how much time in total have you spent doing these activities each day? (In minutes)	18.2 ± 30.4/0 (0–30)	23 ± 24.7/20 (0–30)	0.064	0.32
3a. On how many days in the past week, have you performed STRONG activities for at least 10 continuous minutes, such as running, doing aerobics, playing football, cycling fast, playing basketball, doing housework	0.7 ± 1.8/0 (0–0)	0.7 ± 1.7/0 (0–0)	0.74	0.05
3b. On how many days in the past week, did you do VIGOROUS activities for at least 10 continuous minutes, such as running, doing aerobics, playing football, cycling fast, playing basketball, doing heavy housework at home, in the yard or digging in the gardening, carrying heavy weights or any activity that has greatly increased your breathing or heartbeat. (Say the number of days)	8.6 ± 22.6/0 (0–0)	5.8 ± 14.4/0 (0–0)	0.274	0.28
4a. How much time in total do you spend sitting on a weekday? (in minutes)	335.4 ± 285.9/300 (120–480)	354.1 ± 285.4 /300 (180–480)	0.782	0.03
4b. How much time in total do you spend sitting on a weekend day? (in minutes)	346.5 ± 304.3/300 (120–480)	361.1 ± 286.2/300 (180–480)	0.91	0.0

Note: Data expressed as mean + standard deviation, median (25th percentile - 75th percentile).

The main types of transportation used for the face-to-face consultation were own car (49.0%), Uber (34.2%), and bus (10.8%). Eighty-one participants (82.8%) used to live in the Fortaleza metropolitan area. Thirty-four (34.7%) of the caregivers missed work to attend face-to-face consultations. The average travel time saving was 289.6 ± 177.8 minutes, and money-saving was 106.67 ± 202.50 reais (around USD 18; almost 10% of the current minimum wage in Brazil).

## DISCUSSION

To our best knowledge, this is the first study to assess the feasibility of a telehealth video consultation using WhatsApp for delivering care directly at home to PD patients in Brazil public health system. We provided remote specialized care and health education to PD patients with a 90.7% attendance rate. The low recruitment rate (61.3%) was likely due to a high proportion of patients that could not be reached by their phone number. It is worthy to mention that this study was performed in a public health system located in a poor region of Brazil. Forty-three patients could not be contacted for providing the wrong phone number, or because they changed the phone number, or because their telephones were unable to receive calls. Most likely, the patients did not update their data, or the responsible health professionals did not properly re-register the individuals. The inability of outpatient clinics to get the patients registration data reveals a lack of training of health professionals. Another challenge in the recruitment is due to technical difficulties related to social problems. Some of the study's participants lived in very undeveloped areas or what we call “favelas”, often considered marginalized areas. Due to the absence of governmental investments, these areas lack proper infrastructure, safety, energy, and recreation, providing their residents with a low quality of life. With that being said, the access to technology and internet is very challenging and something not every individual can obtain it. The northeast region had the lowest percentage of residencies with internet access (69.1%). The three most alleged reasons for not using the internet were lack of interest in accessing it, internet access service not being available in the neighborhood, and internet service being expensive. The other reason for the loss of patients in the recruitment–unavailability of caregiver–also shows the Brazilian social problems. 14

The scores in the satisfaction survey were above 80, except for the question on effectiveness (median score of 75): “Do you think video consultations may help improve Parkinson's disease symptoms?” It suggests that a single consultation is insufficient to address all the problems and concerns, especially for those patients that require rehabilitation care with a multidisciplinary approach. 15 The participants were referred to rehabilitation care services, but care was not provided in most cases due to few public rehabilitation centers available. 16 Home care rehabilitation services are scarce in the public health system, especially in the Northeast of Brazil. 17

We found good adherence (54.1%) to non-pharmacological treatment and a higher adherence rate (87.8%) to pharmacological prescription. The World Health Organization (WHO) defines adherence to therapy as “the extent to which a person's behavior—taking medication, following a diet, and/or executing lifestyle changes—corresponds with agreed recommendations from a health care provider 18 ”.

The intervention improved sedentary behavior and the level of physical activity for at least 30 minutes, 3 times a week. Also, the intervention improved perceived sleep duration, sleep disorders, and daytime sleep disturbance. A systematic review of the prevalence of sleep problems during the COVID-19 pandemic showed that their prevalence is high and affects approximately 40% of people from health care populations. 19 Some studies have demonstrated relevant health benefits when the intervention encompasses several behavior changes. 20 21 Some authors have reported better outcomes when behaviors generate synergy and are combined in a single intervention. 22 23 Sedentary lifestyle and sleep problems are highly prevalent in adults and may even have a reciprocal relationship. 23

Our intervention improved the PSQI total score value that changed from 12.1, in the baseline, to 10.7 ( *p* < 0.001) and d = 0.44 (medium effect size), and the subdomain 3 (subjective sleep duration) from 1.7 to 1.1 ( *p* < 0.001) and r = 0.63 (large effect size). The perceived sleep duration improvement observed in our data is most likely related to pharmaceutical treatments such as depression therapy, pain management, sleep disorder management, overactive bladder management, SHE, and physical activity recommendations. Yang et al. 20 conducted a meta-analysis of randomized trials investigating the effects of an exercise training program on sleep quality in middle-aged and older individuals with sleep complaints. According to pooled analyses of the data, exercise training had a moderately favorable influence on sleep quality, as seen by decreases in the overall PSQI score, subdomains of subjective sleep quality, sleep latency, and sleep medicine consumption. Other sleep time characteristics, such as sleep length, efficiency, and disturbance, did not significantly improve. In general practice, SHE is widely used to treat insomnia. According to a meta-analysis conducted by Chung et al. 21 in 2018, SHE was related to sleep improvements based on substantial pre to postintervention changes in PSQI. However, it was less effective than cognitive-behavioral treatment and mindfulness-based therapy. 24

The participants showed good acceptance of virtual consultations despite cultural and technological barriers to digital communications in Brazil. However, some people still have limitations in using the technology, especially older adults, due to the difficulty in learning how to use it, and due to high costs still imposed on access to telephones, and few incentives to digital media. 25 26 Our sample of patients was mostly older adults with low education, which poses a challenge to the feasibility of virtual consultations. 26 Some studies have shown similar results in face-to-face and virtual consultations with good feasibility. 3 27 It is noteworthy that, compared to our sample, the participants in these studies were more educated and reported regular internet use.

Before the COVID-19 pandemic, there was evidence showing that social isolation can affect the physical and mental health of older adults, 28 leading to a higher incidence of depression, in addition to cognitive decline, declining physical performance, and higher mortality. 29 We believe that good adherence to treatment and to a behavior recommendation reflects a positive impact of virtual interactions with our patients during this challenging period of social isolation.

Possibly, the TeleParkinson project was well accepted because our sample study has a mean disease duration of over 10 years and a high proportion of motor complications and hallucinations. Also, the mean SEADL was approximately 70. The modality of the consultations was more suitable for our sample once it prevented the dislocation of patients with severe neurological complications, hallucinations, and low SEADL rates. Van den Bergh et al., 30 in 2021, cited consultations satisfaction and the importance of telemedicine for more severely ill patients with access restriction, as well as palliative patients.

The major limitation of this study is the absence of a control group. Second, our sample comprised mostly participants from the city of Fortaleza and its metropolitan area, and teleconsultation feasibility studies for rural areas are needed. Third, the sample size calculation did not include a power analysis. Fourth, the study involved only single consultation with a geriatrician, and longitudinal patient care is likely to have substantial benefits. Therefore, it is unknown if individuals in this study would have gone to a second telemedicine visit if they had firsthand knowledge of what it entails. About 38.2% of screened patients were not enrolled in the study, which calls the generalizability of patient satisfaction reports into question. Besides, the recruitment of assistant physicians who already care for these patients for teleconsultation does not represent the recruitment of services without this prior involvement.

In conclusion, we indicated that direct-to-home telehealth video consultation using WhatsApp for delivering care to PD patients seems feasible and effective in improving some components of sleep and physical activity level. Further efforts and policy solutions will hopefully make this care model increasingly available for PD patients in Brazil's public health system.
