# Supplementary material for: Telehealth for Parkinson disease patients during the COVID-19 pandemic: the TeleParkinson study
Source: Arq Neuropsiquiatr. 2022 Dec 19;80(10):1026–35. doi: 10.1055/s-0042-1758751 (PMC9770058; doi:10.1055/s-0042-1758751)
Supplement: Supplementary file 1 — Supplementary Material [file 10-1055-s-0042-1758751-s210499.pdf]

Supplementary Table 1. Health education recommendations for patients with mild to moderate Parkinson disease

Subject	Recommendations	Examples with illustrations
Nutrition / Eating habits	- Have at least 3 meals during the day: breakfast, lunch, dinner, and snacks between the breaks.<br>- Eat proteins and dietary fiber.<br>- Avoid processed foods.<br>- Reduce the amount of salt.<br>- Drink 2 L of water per day.	14 fiber-rich foods have been illustrated: avocado, flaxseed, plum, chia, papaya, lettuce, kale, apple, persimmon, chard, watercress, arugula, wheat bran, and oat-meal.<br>Example of proteins: meats (cattle, fish, chicken, lamb), milk, soy, beans, and chickpeas.
Fall preventing	- Wear closed shoes with rubber soles to prevent you from slipping.<br>- Practice physical exercises to develop balance.<br>- Have non-slip mats in the bathroom.<br>- Use handrails when climbing stairs.<br>- Avoid walking on wet floors.<br>- Only take medication prescribed by your doctor.<br>- Avoid animals and obstacles in the pathways of the house.<br>- Do regular evaluations and corrections of hearing and vision to prevent falls.<br>- To avoid crises and the danger of falling, try to empty your bladder every 2 hours.<br>If the issue is no longer under control, wear a diaper or a protector.	Each recommendation was illustrated to enhance learning.
Sleep hygiene	- Regular exercise helps regulate sleep.<br>- Sleep and wake up at the same time during the week as well as on the weekend.<br>- After 6 pm, avoid consuming foods with caffeine.<br>- Avoid napping.	Each recommendation was illustrated to enhance learning.
Physical activity	- Do not exercise on an empty stomach<br>- Exercise when the parkinsonism medication is most effective.<br>- Stretch before you exercise.<br>- Sedentary behavior interruptions: get up to change the station on the television or to grab a drink of water, get up for 2 minutes to every 1 hour, even if you are indoors, try to walk as much as possible.	We suggest exercises such as:<br>- Walking 30 minutes a day.<br>- Sitting and standing in a safe chair (3 × 10 series).<br>- Pelvic lifting while lying down (3 × 10 series).<br>- Kegel exercises.<br>- Hip adduction while lying down with leg lifting (3 × 10 series).<br>- Calf exercises while holding fixed support (3 × 10 series)<br>- Illustrated stretching was given.

Abbreviation: PD, Parkinson disease.

 © 2022. Academia Brasileira de Neurologia. All rights reserved.

Supplementary Table 2. Recommendations for bedridden/chair-bound Parkinson disease patients

Subject	Recommendations	Examples with illustrations
Skin care	- Always check for any redness on the skin. If there are any "cushions" around the area, reduce the body pressure on this part.<br>- Change positions every 2 hours.<br>- Apply moisturizer to the skin, especially in places where you can feel the bone underneath.	
Patient feeding	When you go to eat, keep in mind that you must:<br>- Be in a sitting position (you can use a back pillow for support) and wide awake.<br>- Observe if you are frequently choking. If so, use thickener powder to thicken liquids until they have the consistency of pudding.	- The thickener is a powder; when placed in the liquid, it changes its consistency to resemble gelatin, decreasing the risk of choking when feeding.<br>But if there is frequent choking, a speech therapist should also be consulted.
Exercises	Exercises are essential for staying healthy, and with the help of your caregiver, they can be done in bed or in a wheelchair. You can do these movements twice a day.	We suggest exercises such as:<br>- Move each toe up, down, and rotate.<br>- Hold the ankle and move the foot up, down, sideways, and rotating.<br>- Bend and extend one leg 10 times and then repeat the movement 10 times on the other leg. (This movement can be done in bed or a wheelchair.)<br>- With the person's feet on the bed and the knees bent, do the movement of separating and joining the knees 10 times. In the same position, ask the person to slowly raise and lower the buttocks 10 times.<br>- Raise and lower the person's arms 10 times. Then open and close them.<br>- Make movements with the head forward, backward, and to the sides.
Exercises on a wheelchair		We suggest exercises such as:<br>- Open and close your arms by bringing one hand closer and further away from the other 20 times.<br>- Open and raise your arms 20 times.<br>- Punch upwards with the left hand 10 times and then 10 more times with the right hand. Then do the same thing forward.<br>- Open your arms and rotate forward 5 times and backward 5 times.

Abbreviation: PD, Parkinson disease.

 © 2022. Academia Brasileira de Neurologia. All rights reserved.
